# Chlorine Dioxide Inhibits African Swine Fever Virus by Blocking Viral Attachment and Destroying Viral Nucleic Acids and Proteins

**DOI:** 10.3389/fvets.2022.844058

**Published:** 2022-03-17

**Authors:** Ruiping Wei, Xiaoying Wang, Xiaohong Liu, Chunhe Guo

**Affiliations:** Guangzhou Higher Education Mega Center, State Key Laboratory of Biocontrol, School of Life Sciences, Sun Yat-sen University, Guangzhou, China

**Keywords:** African swine fever (ASF), antiviral, chlorine dioxide, viral attachment, cytokine

## Abstract

African swine fever (ASF) is a highly contagious disease and provokes severe economic losses and health threats. At present no effective vaccine or treatment is available to prevent or cure ASF. Consequently, there is an urgent need to develop effective drugs against ASF virus (ASFV). Chlorine dioxide (ClO_2_), an ideal biocide, has broad-spectrum antibacterial activity and no drug resistance. Here, we found that ClO_2_ strongly inhibited ASFV replication in porcine alveolar macrophages (PAMs). The inhibitory effect of ClO_2_ occurred during viral attachment rather than entry, indicating that ClO_2_ suppressed the early stage of virus life cycle. ClO_2_ showed a potent anti-ASFV effect when added either before, simultaneously with, or after virus infection. Furthermore, ClO_2_ could destroy viral nucleic acids and proteins, which may contribute to its capacity of inactivating ASFV virions. The minimum concentration of degradation of ASFV nucleic acids by ClO_2_ is 1.2 μg/mL, and the degradation is a temperature-dependent manner. These have guiding significance for ClO_2_ prevention and control of ASFV infection in pig farms. In addition, ClO_2_ decreased the expression of ASFV-induced inflammatory cytokines. Overall, our findings suggest that ClO_2_ may be an ideal candidate for the development of novel anti-ASFV prophylactic and therapeutic drugs in swine industry.

## Introduction

African swine fever (ASF) can be considered as one of the most feared epidemic diseases of the pig industry worldwide. ASF is extremely dangerous due to its highly contagious characteristics, high morbidity and mortality rates, extreme resilience to endure high and low temperatures, and ability to be easily spread via a variety of vectors (1). The disease was first described in Kenya in the 1920s and then Outbreaks have been reported periodically outside Africa. In 2007, ASF was introduced into the Caucasus region of Eurasia via the Republic of Georgia. Subsequently, it spread throughout the Caucasus into the Russian Federation, Ukraine, and Belarus (2, 3). In 2018, an outbreak of ASF in pigs was reported in China and then occurred in other Asian countries and regions, causing huge economic losses to the global swine industry (4, 5).

African swine fever virus (ASFV), the etiological agent of ASF, is the sole member of its genus *Asfivirus* and family *Asfarviridae* (*Asfar*, African swine fever and related viruses) and the only known DNA arbovirus (6). The genome of the virus is a linear double-stranded DNA molecule containing around 200 open reading frames and the size of which is about 170–190 kilo base pair depending on the virus strain (7, 8). The virus has a highly genetic and antigenic diversity. To date, there are 24 genotypes based on the viral p72 protein (*B646L* gene), while at least 8 serotypes have been identified by hemadsorption inhibition assay (9). ASFV isolates vary in virulence thus the pathogenetic process of infected animals may be peracute, acute, subacute or chronic (10–12). ASFV is mainly thought to enter the animal body via the upper respiratory tract and can persist in swill and uncooked meat products for several months, thus might be transmitted and spread through the contaminated swine feed or meat products ingested (13). Due to the lack of detailed knowledge concerning the virulence of the virus, viral pathogenesis and immune response to virus infection, and the available cell lines supporting the ASFV replication, there is currently no effective vaccine available to prevent the transmission and spread of ASFV (14, 15). With that in mind, new virus control strategies such as antivirals are urgently needed.

Chlorine dioxide (ClO_2_), a strong oxidant, has a wide application prospect in different fields such as food and environment disinfection, medicine, as well as wastewater or water treatment (16). Most of the disinfectants such as peracetic acid and hydrogen peroxide used nowadays are toxic even at low concentrations (16). However, ClO_2_ has very low toxicity to the animal organism even at adequate antiviral and antibacterial activities (17). Therefore, it is widely used to sterilize the drinking water (18). Due to the property of its chlorination, ClO_2_ can inhibit or destroy microbes such as inactivating bacteria, virus, fungi, parasites and other cellular pathogens (19, 20). We previously demonstrated that ClO_2_ displays potent antiviral and virucidal activities to porcine reproductive and respiratory syndrome virus infection in both Marc-145 cells and porcine alveolar macrophages (PAMs) (21). Previous studies also showed that ClO_2_ is capable of killing feline calicivirus, human herpesvirus, human influenza virus, measles virus, canine parvovirus and human adenovirus (20, 22). However, whether ClO_2_ inhibits ASFV replication remains unknown.

Here, we investigate the anti-ASFV activity of ClO_2_ *in vitro* and address the molecular mechanism of its inhibitory effect on ASFV. We found that ClO_2_ has a great clinical application prospect in the prevention and treatment of ASFV infection in pig industry.

## Materials and Methods

### Cells, Viruses and Compounds

PAMs were isolated from the lungs of 3–8-week-old ASFV-negative piglets (Guangxi State Farms, China) by lung lavage. PAMs were cultured in RPMI-1640 supplemented with 100 U/ml penicillin, 100 μg/ml streptomycin sulfate, and 10% heat-inactivated fetal bovine serum (FBS; PAA, Pasching, Austria). Animals were euthanized and carcasses were treated innocuously. All animal experiments were approved by the Institutional Animal Care and Use Committee of Sun Yat-sen University. ASFV isolate GD19 was propagated and titrated in PAMs and was used throughout the study. ClO_2_ was provided by Guangzhou WellFar Co, Ltd., Guangzhou, China.

### Cytotoxicity Assay

The cytotoxicity of ClO_2_ was tested using the alamarBlue^®^ assay (Invitrogen, USA) according to the manufacturer's instructions. Briefly, PAMs at the density of 1 × 10^5^ per well were seeded in 96-well plates respectively, ClO_2_ at different concentrations were added. Mock-treated cells were set up simultaneously. After incubation for 48 h, 10 μl of alamarBlue^®^ was added to each well and the cultures were incubated for another 3 h. Fluorescence intensity was measured at 570 nm excitation and 590 nm emission wavelengths using a microplate reader (Synergy2, BioTek, USA) and normalized to the control for each sample.

### Quantitative Reverse-Transcription Polymerase Chain Reaction (qRT-PCR)

Total RNA was extracted from PAMs using TRIzol reagent (Magen, China) according to the manufacturer's instructions. Briefly, Reverse Transcription System (A3500, Promega, USA) was used for reverse transcription in 20 μL reaction volume. The reverse-transcription primers were Oligo (dT) 15 primer (C110A, Promega, USA) and Random primer (C118A, Promega, USA). SYBR Green (TaKaRa, Osaka, Japan) real-time PCR was performed using a Light-Cycler 480 PCR system (Roche, Basel, Switzerland). Relative quantities of mRNA accumulation were evaluated using the 2^−Δ*Ct*^ method. The primers used for qRT-PCR are listed in Table 1.

**Table 1:** List of primers used in this study.

**Primer^**a**^**	**Sequence (5^**′−3′**^)^**b**^**	**GenBank number**
**Primers for qRT-PCR**		
p72-F	ACGGCGCCCTCTAAAGGT	MK128995.1
p72-R	CATGGTCAGCTTCAAACGTTTC	
pHPRT1-F	TGGAAAGAATGTCTTGATTGTTGAAG	NM_0010323762
pHPRT1-R	ATCTTTGGATTATGCTGCTTGACC	
IL-6-F	AGAGGCACTGGCAGAAAAC	AF518322.1
IL-6-R	TGCAGGAACTGGATCAGGAC	
IFN-β-F	GCAATTGAATGGAAGGCTTGA	GQ415073.1
IFN-β-R	CAGCGTCCTCCTTCTGGAACT	
**Primers for PCR amplification of ASFV p32 gene and partial p72 gene**		
p32-F	ATGAAAATGG AGGTCATCTT	MK128995.1
p32-R	TAACCATGAGTCTTACCACCTCT	
partial p72-F	ATGCAGCCTA CTCACCACGC	MK128995.1
partial p72-R	AAGTTAATAGCAGATGCCTATACC	

a *F, forward primer; R, reverse primer*.

### Immunofluorescence Assay (IFA)

PAMs were fixed with 4% paraformaldehyde at room temperature (RT) for 15 min and then permeabilized with 0.3% Triton X-100 at RT. After washed three times with PBS, PAMs were blocked with 1% Bovine serum albumin (BSA) in PBS for 30 min at RT, then incubated overnight with an anti-ASFV p30 protein mAb (diluted 1: 200 in PBS; MEDIAN, Republic of Korea) at 4°C and followed by Alexa Fluor^®^ 488-conjugated anti-mouse IgG secondary antibody for 2 h. The nuclei were stained with Hoechst dye 33342 (Sigma-Aldrich, St. Louis, MO, USA). Finally PAMs were examined using fluorescence microscopy (Carl Zeiss, Jena, Germany).

### Western Blot

ASFV-infected or mock-infected PAMs treated with or without ClO_2_ were lysed in RIPA lysis buffer (Beyotime, China), and then electrophoresed onto a 12% SDS-PAGE gel and transferred to a polyvinylidene-fluoride (PVDF) membranes (Roche, USA). The membranes were blocked and incubated with a monoclonal antibody against ASFV p72 protein or GAPDH (Cell Signaling Technology, USA). After washing, membranes were incubated with anti-mouse or anti-rabbit IgG, HRP-conjugated antibody (Cell Signaling Technology, USA). Signal detection was performed using a chemiluminescence reagent (Fdbio Science, China).

### Antiviral Assay

The inhibitory effect of treatment with ClO_2_ on ASFV was analyzed with three different approaches. (I) Pre-treatment: PAMs were pre-treated with ClO_2_ at a concentration of 0.2 μg/mL for 4 h, ASFV at a multiplicity of infection (MOI) of 1 was then added and cells were cultured for another 36 h. (II) Co-treatment: Cells were inoculated with ASFV (MOI = 1) in the presence or absence of ClO_2_ (0.2 μg/mL) for 36 h. (III) Post-treatment: PAMs were infected with ASFV at an MOI of 1 for 4 h at 37°C, and then the viral inoculum was removed and fresh RPMI-1640 medium containing ClO_2_ (0.2 μg/mL) was added and cells were further incubated for 36 h. In all above antiviral assays, the cells were collected for the IFA and qRT-PCR analysis.

### Virus Adsorption Assay

PAMs were cooled at 4°C for 30 min, and then infected with ASFV (MOI = 1) in the presence or absence of ClO_2_ for 3 h at 4°C. After washing three times with ice-cold PBS to remove unbound viral particles, cells were switched to 37°C for a further 4 h. Cells were harvested for IFA and qRT-PCR analysis.

### Virus Entry Assay

Cells were initially infected with ASFV (MOI = 1) for 3 h at 4°C. After binding to cell surface, the viral inoculum was removed and then cells were washed with ice-cold PBS three times and incubated for 4 h at 37°C in the presence or absence of ClO_2_. After washed with PBS three times, cells were harvested to detect ASFV infection by IFA and qRT-PCR.

### Virucidal Assay

ASFV was pre-incubated with various concentrations of ClO_2_ for 2 h at 37°C or pre-incubated with ClO_2_ at the indicated concentration for 1 or 4 h at 37°C. The mixtures were added to PAMs which were then incubated for 2 h at 37°C. The mixtures were finally removed and cells were cultured with RPMI-1640 medium containing 2% FBS for additional 24 h at 37°C, and harvested for the analysis of IFA and qRT-PCR.

### Statistical Analysis

All experiments were performed with at least three independent replicates. Student's *t*-test and one-way ANOVA were used to analyze the data. All data were presented as means ± standard errors (SE). Statistical analysis was performed using SPSS 17.0 and GraphPad Prism 6.0. Differences with *p*-values < 0.05 were considered significant.

## Results

### ClO_2_ Shows a Potent Antiviral Effect on ASFV Infection

**Figure 1:**
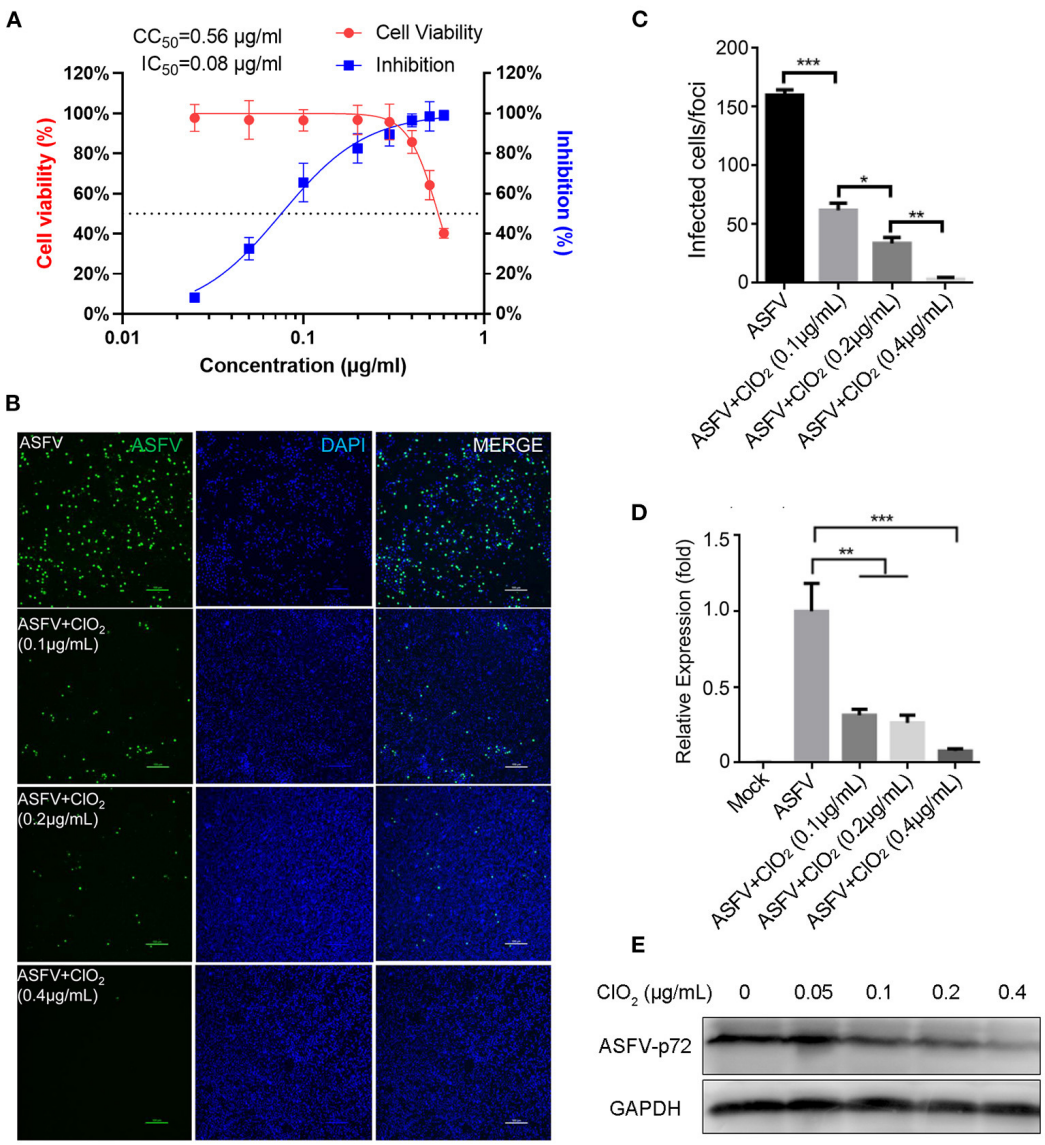
ClO_2_ restrains ASFV replication in a dose-dependent manner in PAMs. **(A)** Dose response curves showing the effect of ClO_2_ on inhibition of ASFV infection (blue) and cytotoxicity (red) in PAMs. The IC_50_ was determined by analyzing the cell infection rate from IFA images, and the CC_50_ was evaluated using the CCK-8 assay. The left and right Y-axis represent virus inhibition (%) and cell viability (%) of ClO_2_, respectively. **(B)** PAMs were infected with ASFV (MOI = 1) in the presence of various concentrations of ClO_2_. After incubation for 24 h, cells were harvested for fluorescence microscope examination. Bar, 100 μm. **(C)** Average number of infected cells per foci in cells treated as B. **(D,E)** PAMs were mock-infected or infected with ASFV at an MOI of 1, and simultaneously co-treated with different concentrations of ClO_2_ for 24 h. Cells were then collected for the detection of viral p72 mRNA **(D)** and protein **(E)** levels by qRT-PCR and western blot analysis, respectively. Data are representative of the results of three independent experiments (means ± SE). Significant differences compared with control group are denoted by *P < 0.05, **P < 0.01, and ***P < 0.001.

**Figure 2:**
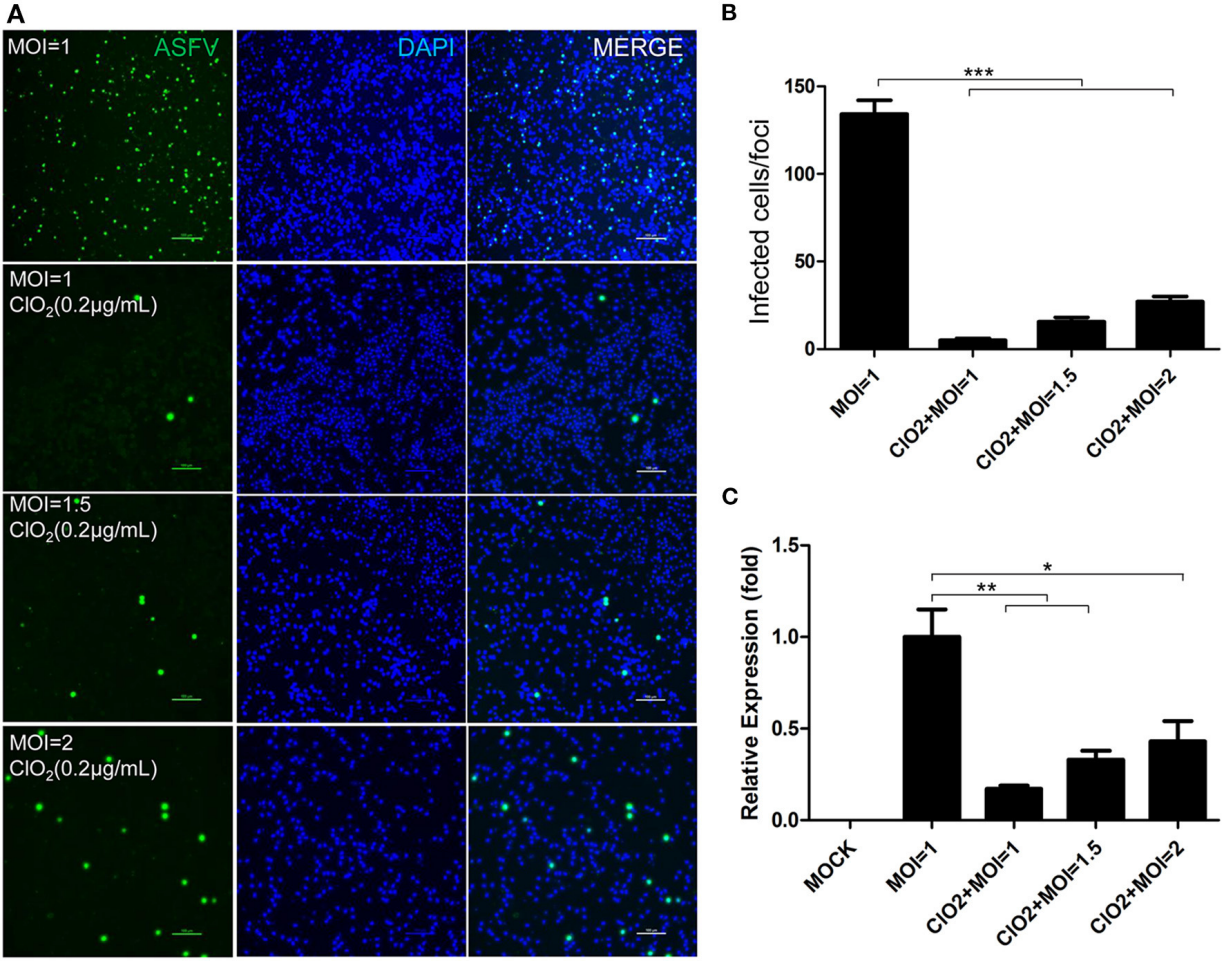
ASFV at different MOIs is inhibited by ClO_2_ in PAMs. **(A)** Cells were inoculated with ASFV at different MOIs in the presence or absence of ClO_2._ Cells were then cultured for 24 h and IFA was performed to evaluate the viral p30 expression. Bar, 100 μm. **(B)** Average number of infected cells per foci. **(C)** PAMs were mock-infected or infected with ASFV at various MOIs, and simultaneously co-treated with ClO_2_ at the indicated concentration. At 24 hpi, cells were measured by qRT-PCR for the determination of the transcription level of viral p72 gene. Data are representative of the results of three independent experiments (means ± SE). Significant differences compared with control group are denoted by *P < 0.05, **P < 0.01, and ***P < 0.001.

To examine the inhibitory effect of ClO_2_ on ASFV replication, the half maximal inhibitory concentration (IC_50_) and half maximal cytotoxic concentration (CC_50_) values of ClO_2_ were assessed using a non-linear regression model in GraphPad Prism 9.0. As shown in Figure 1A, ASFV infection was inhibited by ClO_2_ at an IC_50_ of 0.08 μg/ml, as determined by analyzing the cell infection rate from IFA images, and the CC_50_ value of ClO_2_ measured using the alamarBlue^®^ assay was calculated to be 0.56 μg/ml in PAMs. We next performed IFA and qRT-PCR assays to determine the effect of ClO_2_ on ASFV, the concentration of ClO_2_ used in subsequent experiments was non-cytotoxic to PAMs. Upon ClO_2_ treatment, ASFV p30-specific staining was remarkably decreased in a dose-dependent manner in PAMs compared to that in mock-treated cells following ASFV infection (Figure 1B), suggesting that the infection and the spread of the virions to the neighboring cells are blocked by ClO_2_. The number of infected cells was notably diminished with ClO_2_ treatment (Figure 1C). Consistently, the mRNA and protein levels of the viral p72 were significantly reduced by ClO_2_ treatment compared with the mock-treated cells post ASFV infection, and the inhibition pattern is also in a dose-dependent manner (Figures 1D,E). To further test its antiviral activity on ASFV replication, PAMs were infected with ASFV at different MOIs (at MOIs of 1, 1.5 or 2) in the presence or absence of ClO_2_ (0.2 μg/mL). As shown in Figure 2, consistent with these findings above, ClO_2_ significantly suppressed the viral infection and replication in PAMs infected with ASFV at various MOIs.

**Figure 3:**
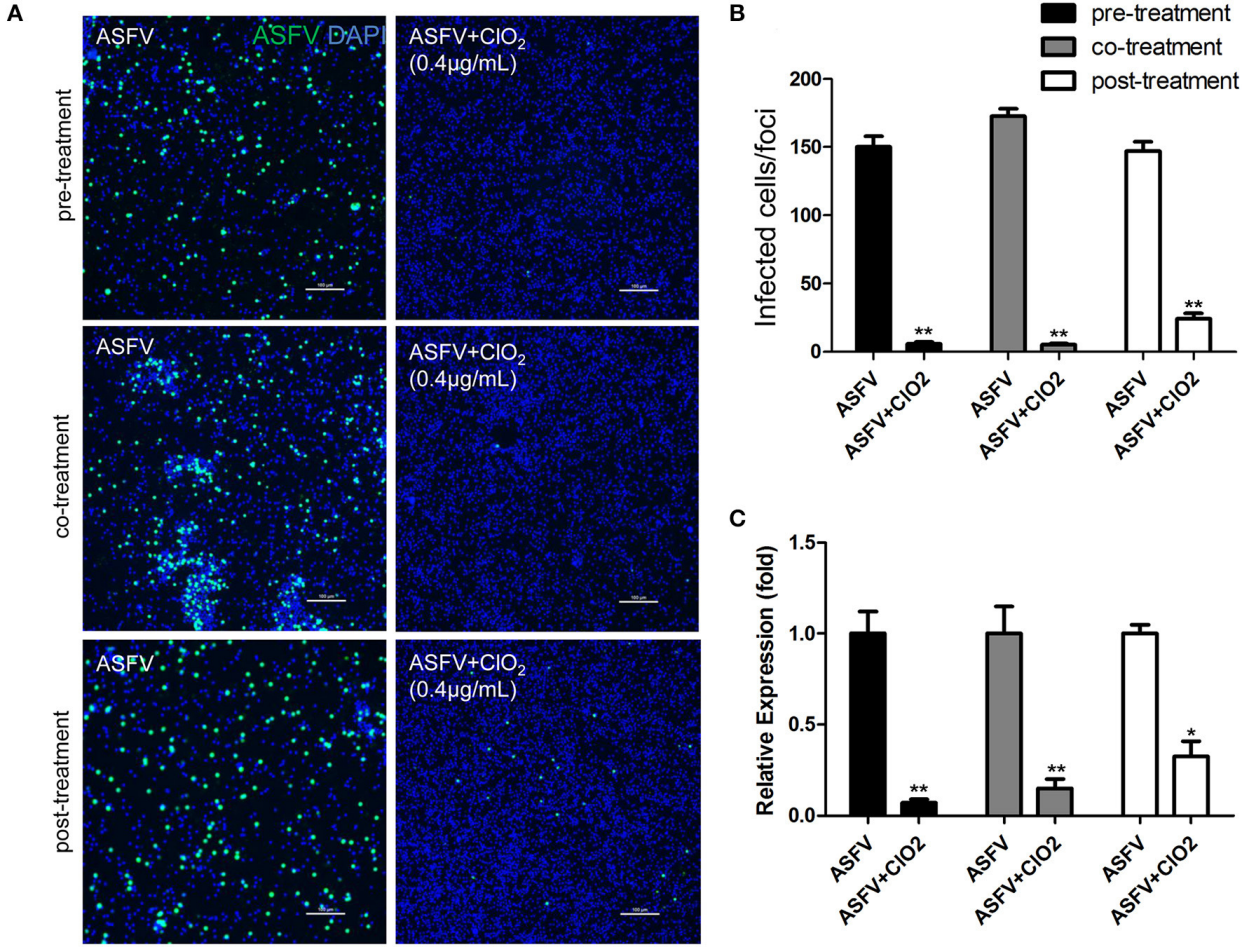
ClO_2_ shows effective antiviral activity against ASFV regardless of pre-treatment, co-treatment or post-treatment. **(A,C)** ClO_2_ treatment was performed either before (pre-treatment), simultaneously with (co-treatment), or after ASFV infection (post-treatment) in PAMs. After cultured for 24 h, cells were fixed for the determination of the expression of virus p30 protein using fluorescence microscope **(A)** or harvested for the evaluation of the mRNA expression of the viral p72 by qRT-PCR **(C)**. Bar, 100 μm. **(B)** Average number of infected cells per foci. Data are representative of the results of three independent experiments (means ± SE). Significant differences compared with control group are denoted by *P < 0.05, **P < 0.01, and ***P < 0.001.

Since the order in which the virus and the compound are added to the cells may affect the antiviral effect of the compound, we therefore assessed the effects of ClO_2_ treatment on ASFV replication using three different approaches as described in the Materials and Methods. As shown in Figure 3, ClO_2_ displayed a strong antiviral activity both at the protein level of viral p30 and at the transcription and protein levels of viral p72 when administered with either the pre-, co-, or post-treatment approach. Taken together, these data demonstrate that ClO_2_ shows a powerful antiviral effect on ASFV infection in PAMs *in vitro*.

### ClO_2_ Shows a Potent Extracellular Virucidal Activity Against ASFV Infection

**Figure 4:**
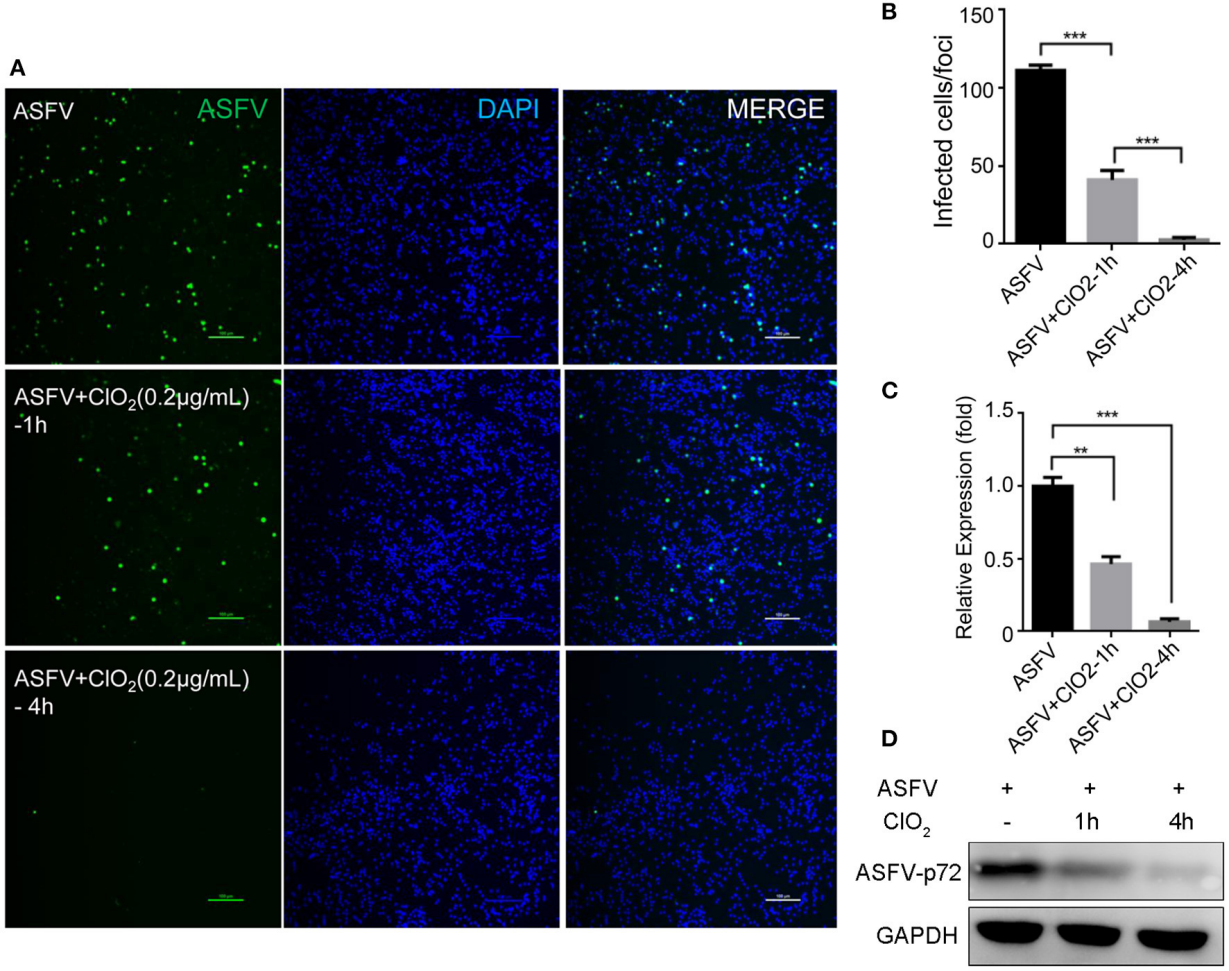
ClO_2_ displays a strong extracellular virucidal effect on ASFV. **(A,C,D)** ClO_2_ at the concentration of 0.2 μg/mL was pre-incubated with ASFV (MOI = 1) for 1 or 4 h at 37°C, and then the mixtures were diluted and added to PAMs for 2 h at 37°C. Finally the mixtures were removed and cells were replenished with fresh RPMI-1640 supplemented with 2% FBS and cultured for additional 24 h. IFA was performed to detect the expression of viral protein using an anti-ASFV p30 mAb **(A)**. The mRNA and protein levels of virus p72 were examined by qRT-PCR **(C)** and western blot **(D)** analysis, respectively. Bar, 100 μm. **(B)** Average number of infected cells per foci. Data are representative of the results of three independent experiments (means ± SE). Significant differences compared with control group are denoted by **P < 0.01 and ***P < 0.001.

**Figure 5:**
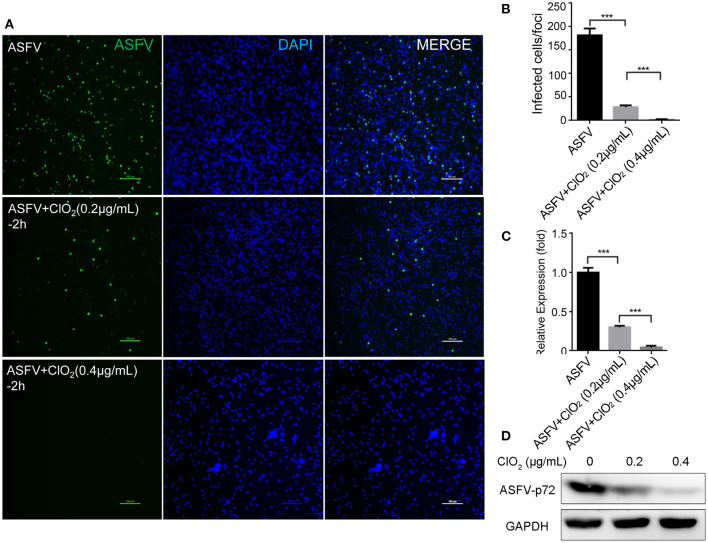
ClO_2_ inactivates ASFV virions in a dose-dependent manner. **(A,C,D)** ASFV was pre-incubated with various concentrations of ClO_2_ for 2 h at 37°C. The mixtures were then added to PAMs. After incubation for 2 h at 37°C, cells were supplied with fresh RPMI-1640 medium containing 2% FBS and cultured for another 24 h at 37°C. Cells were finally harvested for the analysis of IFA **(A)**, qRT-PCR **(C)** and western blot **(D)**. Bar, 100 μm. **(B)** Average number of infected cells per foci. Data are representative of the results of three independent experiments (means ± SE). Significant differences compared with control group are denoted by ***P < 0.001.

Previous studies have shown that ClO_2_ has great ability to inactivate bacteria, virus, fungi, parasites and other cellular pathogens (23–25). To demonstrate that whether ClO_2_ can kill the ASFV *in vitro*, a virucidal assay was performed on PAMs. As shown in Figure 4, a lot of fluorescence representing viral burden in visual field was observed following the treatment of ASFV only using a fluorescence microscope. However, the viral fluorescence was dramatically reduced in ASFV-infected cells upon ClO_2_ treatment. The decline was even more pronounced when the virus was incubated with ClO_2_ for 4 h at 37°C. Consistently, the mRNA expression of the viral p72 was notably repressed in the presence of ClO_2_ treatment for 1 or 4 h (Figure 4C). Similar phenomenon was also observed when the virus was incubated with different concentrations of ClO_2_ for 2 h (Figure 5). Overall, ClO_2_ can effectively inactivate the ASFV virions *in vitro*.

### ClO_2_ Inhibits Viral Attachment but Not Entry

**Figure 6:**
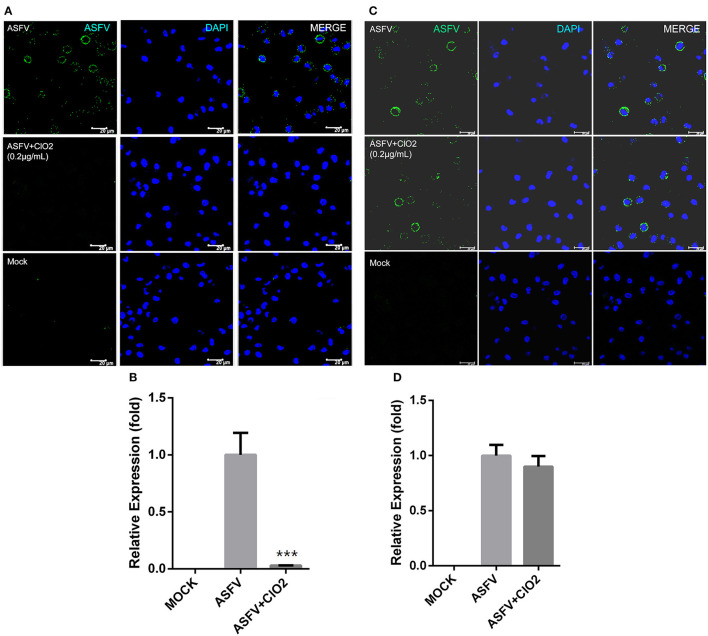
ClO_2_ inhibits virus adsorption rather than entry during ASFV life cycle. **(A,B)** Cells were pre-chilled for 30 min at 4°C and mock-infected or infected with ASFV (MOI = 1) in the presence or absence of ClO_2_. After cultured for 3 h at 4°C, cells were then washed and switched to 37°C for additional 4 h. The inhibitory effect was determined by IFA **(A)** and qRT-PCR **(B)** assays. Bar, 20 μm. **(C,D)** For the viral entry assay, cells were cooled for 30 min at 4°C and mock-infected or infected with ASFV at an MOI of 1 for 3 h at 4°C. Cells were then washed three times to remove unbound virions and incubated at 37°C in the presence or absence of ClO_2_ for 4 h. Cells were fixed and stained for ASFV p30 protein using fluorescence microscope **(C)** or collected to determine the mRNA level of viral p72 protein **(D)**. Bar, 20 μm. Data are representative of the results of three independent experiments (means ± SE). Significant differences compared with control group are denoted by ***P < 0.001.

Viral infection of target cells involves the following processes: adsorption, entry, and uncoating. To characterize the molecular mechanism of anti-ASFV activity of ClO_2_ and to identify which stage of the viral life cycle is suppressed by ClO_2_, a viral adsorption assay described in Materials and Methods was first performed to test whether ClO_2_ is able to inhibit ASFV attachment to PAMs. As shown in Figures 6A,B compared with the mock-treated cells inoculated with ASFV (MOI = 1), the translation level of viral p30 protein and the transcription level of viral p72 were sharply decreased in infected cells in the presence of ClO_2_ during the period of virus adsorption. The data indicate that ClO_2_ is capable of diminishing the quantity of infectious virions attached to cell surface. We next investigated whether ClO_2_ can restrain the entry phase of virus life cycle (the protocol was described in Materials and Methods). The ASFV p30-specific staining which represents the virus yield in virus-infected cells with the treatment of ClO_2_ was comparable to that of cells following the treatment of ASFV only, during the entry of the virus (Figure 6C). The level of mRNA expression of the viral p72 protein showed a similar phenomenon (Figure 6D). Taken together, these data indicate that ClO_2_ shows a potent inhibitory effect on the viral adsorption rather than the internalization process of ASFV life cycle in PAMs.

### The ASFV Inactivation by ClO_2_ Treatment May Be Attributed to Its Effects on Viral Nucleic Acids and Proteins

**Figure 7:**
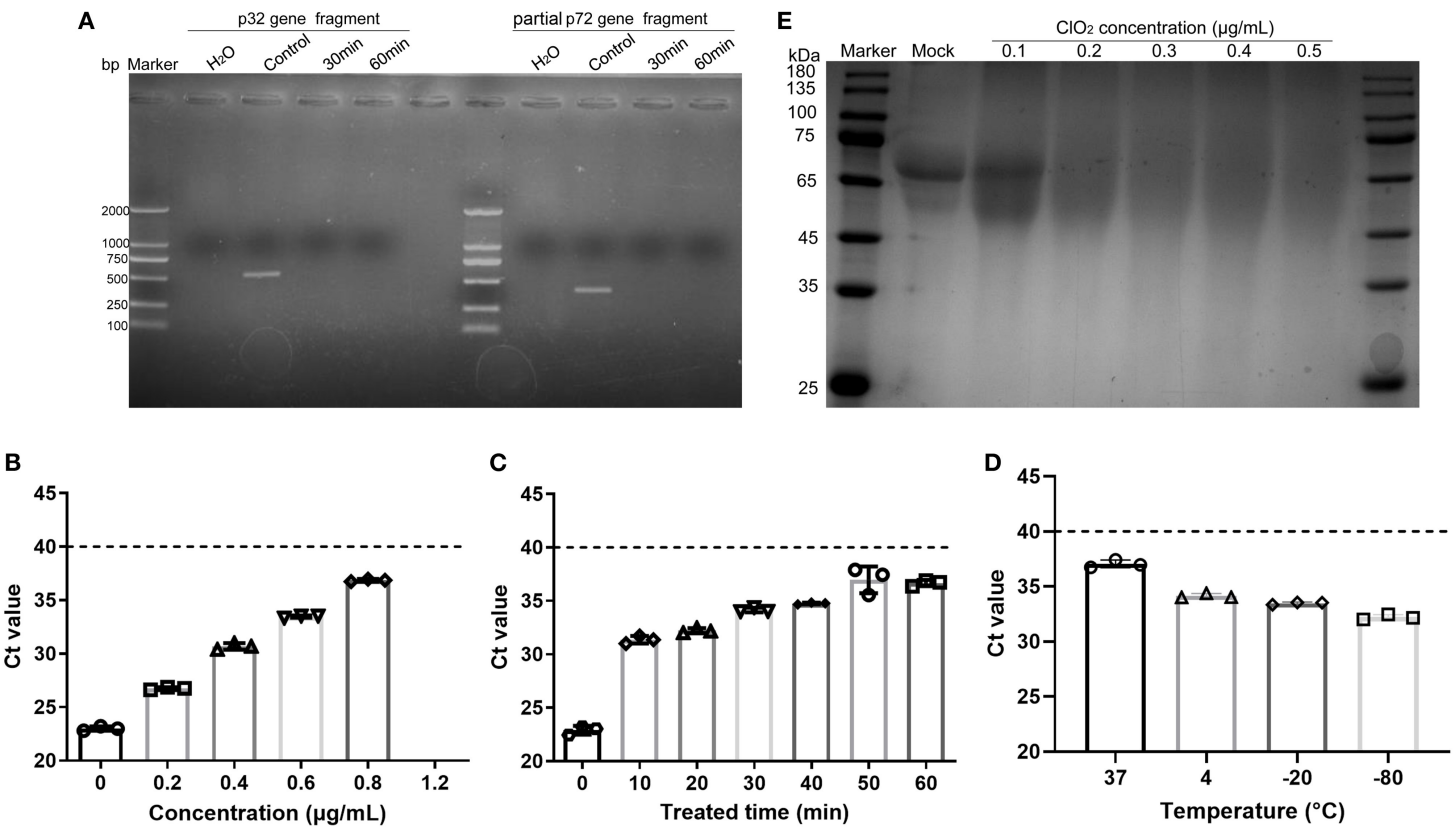
ClO_2_ inactivation of ASFV virions may due to its effects on viral nucleic acids and proteins. **(A)** ASFV was incubated with ClO_2_ at the indicated concentration for 30 or 60 min at 37°C, the fragments of virus p32 gene (558 bp) and partial p72 gene (478 bp) were amplified by PCR to test the viral nucleic acids damage by ClO_2_. The starting amount of ASFV added to each reaction is 5.8 × 10^6^ copies. **(B–D)** To determine the minimum concentration at which ClO_2_ destroys the viral nucleic acids and the effect of ClO_2_ in killing ASFV in various temperature, different concentrations of ClO_2_ were incubated with ASFV for 30 min at 37°C **(B)**, or ClO_2_ at the indicated concentration was incubated with the virus at 37°C for various times **(C)** or at different temperature for 60 min **(D)**, the mixtures were then collected and quantitative PCR was performed to measure the level of viral p72 gene. The threshold cycle (Ct) values which are below the cut-off (dashed line) mean ASFV-positive. **(E)** The effects of ClO_2_ on the viral proteins were also tested. ClO_2_ at different concentrations was incubated with ASFV for 30 min at 37°C, the mixtures were subjected to SDS-PAGE analysis. Data are representative of the results of three independent experiments (means ± SE).

Previous studies have shown that ClO_2_ produces its antiviral activity against hepatitis A virus by acting on viral nucleic acids and proteins (19, 26). Since ClO_2_ has the ability to kill ASFV, we next investigated that whether ClO_2_ can damage the nucleic acids of the virus. ClO_2_ at the concentration of 0.1 μg/mL was incubated with ASFV for 30 or 60 min at 37°C, and then the mixtures were harvested for viral nucleic acids analysis by PCR. The starting amount of ASFV added to each reaction is 5.8 × 10^6^ copies. As shown in Figure 7A, there were no detectable fragments of viral p32 gene and partial p72 gene upon ClO_2_ treatment, indicating that the ASFV nucleic acids can be destroyed by ClO_2_ treatment. To determine the minimum concentration at which ClO_2_ destroys the viral nucleic acids, quantitative PCR was performed (27). As shown in Figure 7B, ClO_2_ at the concentration of 1.2 μg/mL completely destroyed the viral genome (no Ct values were detectable). The viral nucleic acids were almost degraded by ClO_2_ treatment at the concentration of 0.2 μg/mL for 60 min at 37°C (Figure 7C). Since the effect of ClO_2_ in killing ASFV virions is also affected by temperature, we next investigate the ability of viral nucleic acids damage by ClO_2_ in different temperatures. As expected, the Ct values decreased with the drop of temperature post ClO_2_ treatment (Figure 7D), indicating that ClO_2_ degrades ASFV nucleic acids in a temperature-dependent manner. The effect of ClO_2_ on the viral proteins was also tested. ClO_2_ at different concentrations was incubated with ASFV for 30 min at 37°C. As expected, ASFV was degraded with ClO_2_ treatment, and the minimum concentration of degradation is 0.2 μg/mL (Figure 7E). Taken together, ClO_2_ is capable of damaging the ASFV nucleic acids and degrading the viral proteins, which may contribute to its inactivation of ASFV.

### ClO_2_ Decreases the Level of Inflammatory Cytokines Induced by ASFV

**Figure 8:**
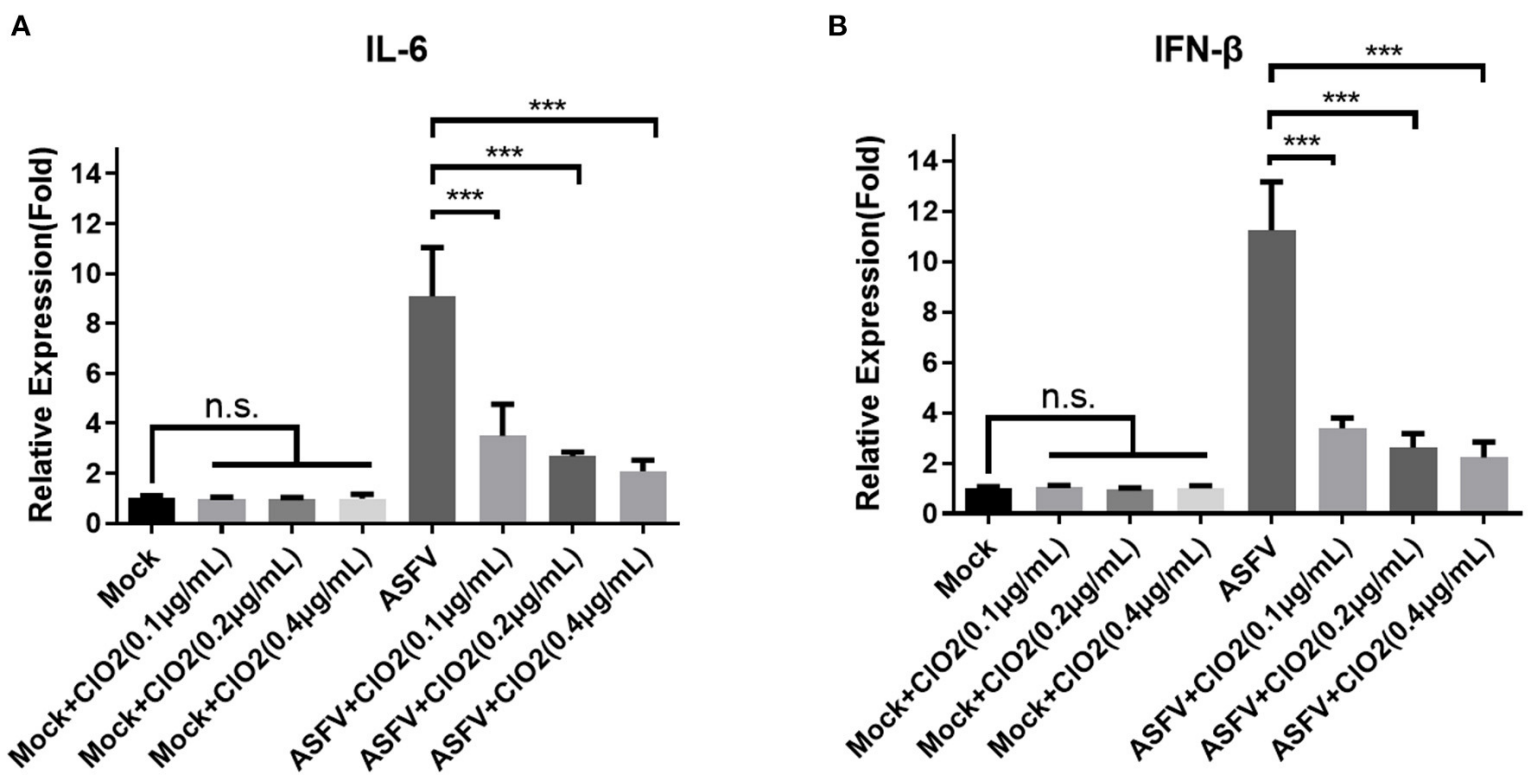
Cytokines expression in PAMs treated with ClO_2._ **(A,B)** PAMs were mock infected or infected with ASFV at an MOI of 1 in the presence of various concentrations of ClO_2_. After cultured for 6 h, cells were lysed for the determination of the expression of IL-6 **(A)** and IFN-β **(B)** using qRT-PCR. Data are representative of the results of three independent experiments (means ± SE). Significant differences compared with control group are denoted by ***P < 0.001.

Since PAMs are immune cells and play an important role in the innate immune response, we next investigated the effects of ClO_2_ on the expression of cytokines such as interleukin-6 (IL-6) and interferon-β (IFN-β), which are known to be related to the host antiviral and inflammatory reactions. As shown in Figure 8, the upregulation of IL-6 and IFN-β induced by ASFV infection was significantly diminished in the presence of ClO_2_ in PAMs at 6 hpi. The expression level of IL-6 and IFN-β in cells treated with ClO_2_ alone was comparable to that of mock-treated cells. These data suggest that ClO_2_ inhibition of cytokines expression caused by ASFV may contribute to its capacity of suppressing virus replication.

## Discussion

Chinese pig farming accounts for 60% of the global pig production industry. The first ASF case in China was reported in August 2018 and the virus has rapidly spread throughout the whole country, leading to serious economic losses to the pig industry in China (4). At present no effective vaccine or treatment is available to prevent or cure ASF, the only possible way to control this disease is the implementation of strict biosecurity measures, and the continuing threat of the disease is overwhelming (28, 29). Therefore, there is an urgent need to develop effective drugs against the virus.

Previous researches refer to ClO_2_ as an ideal biocide (16). ClO_2_ has a broad spectrum antimicrobial activity, and it needs only a few milliseconds to kill bacteria. Although the disinfectants such as sodium hypochlorite and hydrogen peroxide currently in use show an anti-microorganism effect, they are toxic even at low concentrations. However, ClO_2_ has little toxicity to humans and animals even at adequate pathogens inactivating activity. In addition, the main current health challenges are the rise of drug-resistant bacteria and the decline of effective new antibiotics. Since ClO_2_ damages pathogens via destroying nucleic acids and key proteins, the pathogens cannot resist ClO_2_ through any of their resistance mechanisms (16). Therefore, due to its broad-spectrum antimicrobial activity, and non-drug resistance side effects, ClO_2_ is widely used in many fields such as drinking water, fruits in food industry, and environment disinfection. In this study, we demonstrated that ClO_2_ could effectively inhibit ASFV infection and replication in PAMs (Figures 1–3) and inactivate ASFV by eliminating viral nucleic acids and proteins (Figure 7). To demonstrate that ClO_2_ is not a PCR inhibitor, we amplified the housekeeping gene GAPDH in Marc-145 cells in the presence or absence of ClO_2_. No changes were observed in the amplification efficiency of GAPDH upon ClO_2_ treatment (data not shown), suggesting that ClO_2_ indeed directly destroys ASFV genomic DNA.

If the purity of ClO_2_ is low and contains impurities, its use is harmful to the human body and food (18). In this study, to maximize the safety of ClO_2_ and to eliminate or reduce the impurities, we used ClO_2_ with a purity of 99%, and ClO_2_ is dissolved in water. Due to the volatilization of the gas, the residence time of ClO_2_ in aqueous solution is very short.

Inactivation of infectivity is the most important and direct index to evaluate the effects of virus disinfection (19). Here, we showed that ClO_2_ could inactivate the ASFV virions which may be due to its destructive effects on viral nucleic acids and proteins (Figures 4, 5, 7). Furthermore, we found that the minimum concentration of degradation of ASFV nucleic acids by ClO_2_ was 1.2 μg/mL (Figure 7B), which has guiding significance for ClO_2_ prevention and control of ASFV infection in pig farms. In addition, we showed that ClO_2_ treatment caused a reduction of cytokines expression induced by ASFV (Figure 8), which might contribute to its suppression of the virus. However, whether *in vivo* tests in pigs show consistent results needs further study.

In conclusion, our study proves for the first time that ClO_2_, the purity of which is 99%, has very little cytotoxicity and exhibits a very strong anti-ASFV activity via targeting attachment process of viral life cycle. ClO_2_ has the ability to damage viral nucleic acids and proteins to inactivate ASFV virions. Overall, ClO_2_ has the potential to develop into a novel drug against ASFV infection in swine industry in the future. The *in vivo* anti-ASFV effect of ClO_2_ on pigs requires further testing.

## Data Availability Statement

The original contributions presented in the study are included in the article, further inquiries can be directed to the corresponding authors.

## Ethics Statement

The animal study was reviewed and approved by the Institutional Animal Care and Use Committee of Sun Yat-sen University.

## Author Contributions

CG: conceptualization, formal analysis, resources, writing—review and editing, visualization, and project administration. RW and XW: methodology and software. RW, XW, and CG: validation. XL and CG: investigation, data curation, supervision, and funding acquisition. RW: writing—original draft preparation. All authors have read and agreed to the published version of the manuscript.

## Funding

This work was jointly supported by National Natural Science Foundation of China (32072695 and 31872329), Natural Science Foundation of Guangdong province (2019B1515210024), and a special grant (Grant No. 2019B020211003) for detection of ASFV from Guangdong Provincial Department of Science and Technology.

## Conflict of Interest

The authors declare that the research was conducted in the absence of any commercial or financial relationships that could be construed as a potential conflict of interest.

## Publisher's Note

All claims expressed in this article are solely those of the authors and do not necessarily represent those of their affiliated organizations, or those of the publisher, the editors and the reviewers. Any product that may be evaluated in this article, or claim that may be made by its manufacturer, is not guaranteed or endorsed by the publisher.
