# Corrigendum: Chlorine Dioxide Inhibits African Swine Fever Virus by Blocking Viral Attachment and Destroying Viral Nucleic Acids and Proteins

**DOI:** 10.3389/fvets.2022.937653

**Published:** 2022-06-09

**Authors:** Ruiping Wei, Xiaoying Wang, Xiaohong Liu, Chunhe Guo

**Affiliations:** Guangzhou Higher Education Mega Center, State Key Laboratory of Biocontrol, School of Life Sciences, Sun Yat-sen University, Guangzhou, China

**Keywords:** African swine fever (ASF), antiviral, chlorine dioxide, viral attachment, cytokine

In the published article, there was an error regarding the list of authors. The authors “Yongchang Cao, Lang Gong and Guihong Zhang” should be excluded in the published article. The corrected Author Contributions statement appears below.

## Author Contributions

CG: conceptualization, formal analysis, resources, writing—review and editing, visualization, and project administration. RW and XW: methodology and software. RW, XW, and CG: validation. XL and CG: investigation, data curation, supervision, and funding acquisition. RW: writing—original draft preparation. All authors have read and agreed to the published version of the manuscript.

The authors apologize for this error and state that this does not change the scientific conclusions of the article in any way. The original article has been updated.

## Publisher's Note

All claims expressed in this article are solely those of the authors and do not necessarily represent those of their affiliated organizations, or those of the publisher, the editors and the reviewers. Any product that may be evaluated in this article, or claim that may be made by its manufacturer, is not guaranteed or endorsed by the publisher.

